# AI in scientific writing and publishing: A call for critical engagement

**DOI:** 10.1192/j.eurpsy.2025.10061

**Published:** 2025-07-04

**Authors:** Sophia Frangou, Umberto Volpe, Andrea Fiorillo

**Affiliations:** 1 Department of Psychiatry, Icahn School of Medicine at Mount Sinai, New York, NY, USA; 2 Djavad Mowafadgian Centre for Brain Health, University of British Columbia, Vancouver, BC, Canada; 3 School of Specialization in Psychiatry, Marche Polytechnic University, Ancona, Italy; 4 Department of Psychiatry, University of Campania Luigi Vanvitelli, Caserta, Italy

**Keywords:** artificial intelligence, guidance, scientific writing, scientific publishing

## Abstract

Artificial intelligence (AI) is transforming nearly every domain of science, and scholarly publishing is no exception. From automated language editing to machine-assisted peer review and large-scale content analysis, AI tools are increasingly embedded in scientific writing and publishing. The response from the scientific community has ranged from cautious optimism to outright skepticism. This viewpoint aims to articulate an editorial perspective on the integration of AI into scientific writing and publishing, evaluating both the opportunities and tensions that arise, and offering principles for navigating the road ahead.

Artificial intelligence (AI) is transforming nearly every domain of science, and scholarly publishing is no exception. From automated language editing to machine-assisted peer review and large-scale content analysis, AI tools are increasingly embedded in scientific writing and publishing. The response from the scientific community has ranged from cautious optimism to outright skepticism. This viewpoint aims to articulate an editorial perspective on the integration of AI into scientific writing and publishing, evaluating both the opportunities and tensions that arise, and offering principles for navigating the road ahead.

## The promise of AI for scientific publishing

Artificial intelligence has introduced new possibilities for managing the operational and intellectual demands of scientific publishing. As submission volumes continue to rise across nearly all disciplines, editors face increasing pressure to ensure that decisions are not only timely and efficient, but also fair, consistent, and scientifically robust. AI applications, when implemented thoughtfully, offer a range of tools that can enhance the editorial process at multiple stages – from initial screening to final decision-making – without displacing the critical role of human judgment.

One of the earliest and most widely adopted uses of AI in editorial workflows has been the automation of manuscript screening. Platforms such as iThenticate (https://www.ithenticate.com/) and Proofig (https://www.proofig.com/) enable editorial teams to rapidly detect plagiarism, image manipulation, and other forms of scientific misconduct. These tools function by comparing submitted content against vast repositories of published material, flagging instances of textual duplication or image irregularity with speed and precision difficult to match through manual review. In parallel, language-checking systems powered by natural language processing, such as Writefull (https://writefull.com/) and Grammarly Business (https://www.grammarly.com/business) are increasingly used by editorial offices to identify grammatical inconsistencies, unclear phrasing, or non-standard usage prior to peer review, thereby improving the baseline quality of submissions and reducing reviewer burden.

AI has also introduced new efficiencies in the triage phase of editorial assessment. Semantic analysis engines, including StatReviewer (https://www.statreviewer.com/) and Elsevier’s AIRA platform (https://www.elsevier.com/editors/aira), are capable of evaluating the content of a manuscript relative to the journal’s thematic scope, as well as predicting its potential citation impact. These systems synthesize information from abstracts, keywords, and references to assess alignment with editorial priorities, helping editors distinguish high-potential submissions from those that are less likely to advance through review. Such tools are particularly useful in high-throughput environments, where editors must make preliminary assessments within constrained timeframes.

Reviewer identification is another domain where AI has proven increasingly valuable. Traditionally, a time-intensive and often opaque process, reviewer matching can now be supported by platforms like Reviewer Finder (https://reviewerfinder.springernature.com/) and JANE (Journal Author Name Estimator; https://jane.biosemantics.org/). These systems use machine learning algorithms to scan publication databases and match manuscripts with peer reviewers based on shared topical focus, methodological expertise, and availability. By facilitating quicker and more accurate reviewer assignments, these tools help accelerate time to first decision – a particularly pressing metric in fields characterized by rapid discovery cycles, such as biomedical sciences or artificial intelligence itself.

Emerging applications of AI are also transforming how editors interpret and synthesize manuscript content. Tools like SciSummary (https://scisummary.com/) and Scholarcy (https://www.scholarcy.com/) use large language models to generate concise summaries of a manuscript’s objectives, methods, and findings. These summaries can assist editors in forming an initial appraisal of complex or interdisciplinary submissions, particularly when content falls outside their core area of expertise. Some editorial platforms have begun integrating proprietary summarization engines to generate editorial digests that accompany reviewer reports or decision letters, with the goal of standardizing communication and enhancing transparency in editorial decisions.

Importantly, the potential efficiency benefits of these AI-enabled tools extend across journals of varying scale. High-volume publications may use AI to manage the logistical burden of thousands of submissions, while smaller or resource-constrained journals may rely on AI to compensate for limited staff capacity. In both cases, AI can serve as a force multiplier, expanding what editorial teams can accomplish without compromising the centrality of human discretion and responsibility.

## The promise of AI for scientific writing

AI tools are also reshaping the experience of authorship in meaningful and increasingly consequential ways. For researchers, particularly early-career investigators, scholars working in under-resourced institutions, or those writing in a non-native language, the path to publication often presents both linguistic and procedural obstacles. AI-powered applications offer a suite of tools that, when used judiciously, can enhance the quality, efficiency, and inclusivity of the scholarly communication process.

Reference management systems such as EndNote (https://click.endnote.com/) and Zotero (https://www.zotero.org/) have long served as essential infrastructure in academic writing, facilitating the organization of bibliographies and citation formatting. In recent years, these platforms have incorporated AI-enhanced capabilities that go beyond reference storage. Tools like EndNote Click, Connected Papers (https://www.connectedpapers.com/), and ResearchRabbit (https://www.researchrabbit.ai/), Consensus (https://consensus.app/), Elicit (https://elicit.org/), and SciteAI (https://scite.ai/) can assist authors in identifying relevant literature and conceptual linkages within the existing body of work. These features help authors situate their contributions more precisely and may also enhance the citation relevance and theoretical coherence of submitted manuscripts.

Among the most transformative developments is the application of AI to language editing. Platforms such as ChatGPT (https://openai.com/chatgpt), Writefull, and DeepL Write (https://www.deepl.com/write) provide immediate, context-sensitive feedback on grammar, syntax, and stylistic clarity. Unlike traditional editing services, these tools are accessible at scale and at low cost, making them particularly valuable for scholars from non-English-speaking regions who may encounter linguistic bias during peer review. By lowering language-related barriers, such technologies can contribute to a more equitable publishing environment where scientific merit, rather than linguistic fluency, is the primary basis for editorial assessment. Moreover, a growing number of tools now offer deeper support for academic writing.

AI is also reshaping how authors navigate the landscape of journal selection and submission strategy. Most major publishers have developed proprietary tools to guide authors in identifying appropriate journals within their own portfolios. For example, Elsevier’s Journal Finder (https://journalfinder.elsevier.com/), Springer Nature’s Journal Suggester (https://journalsuggester.springer.com/), and Wiley’s Journal Finder (https://journalfinder.wiley.com/) analyze a manuscript’s title, abstract, and keywords to recommend journals based on scope alignment, citation metrics, and historical acceptance patterns. These tools are useful for ensuring internal consistency between manuscript content and journal remit, potentially reducing desk rejections and shortening time to decision. Complementing these are independent, cross-publisher services that offer broader and often more flexible guidance. Tools such as JANE (Journal/Author Name Estimator) and the Edanz Journal Selector (https://www.edanz.com/journal-selector) evaluate textual inputs against large databases of published articles to recommend suitable journals across multiple publishing platforms. JANE, for example, leverages Medline-indexed content to suggest outlets in the biomedical sciences, ranking options by textual similarity to existing literature. Edanz integrates citation metrics, turnaround times, and editorial policies to help authors align their strategic goals – whether visibility, speed, or likelihood of acceptance – with the most appropriate publishing venues. By offering recommendations across publishers, these tools expand the range of submission options and empower authors to make more informed choices.

Together, these developments underscore the growing role of AI not just in facilitating scientific writing but in reshaping how authors engage with the intellectual and procedural demands of academic publishing.

## Epistemic and ethical risks

While the integration of AI into manuscript preparation and editorial workflows holds considerable promise, it also introduces risks across two interrelated but conceptually distinct domains: epistemic and ethical.

Epistemic risks pertain to the credibility, fairness, and reliability of the knowledge that scientific publishing seeks to disseminate. They involve questions about how AI shapes judgments of scholarly merit. For editors, the most immediate epistemic concern lies in the potential for AI tools to amplify existing biases in manuscript evaluation and peer review. For instance, AI-based triage systems trained on historical publication data may privilege research topics, institutional affiliations, or geographic origins that reflect entrenched norms, thereby narrowing the epistemic diversity of the literature. Similarly, reviewer recommendation algorithms may repeatedly prioritize reviewers from overrepresented networks, failing to recognize qualified experts from underrepresented regions or disciplines. Such tendencies risk reproducing knowledge hierarchies under the guise of algorithmic objectivity.

Editors must also grapple with the reproducibility and consistency of AI-assisted decisions. It is not uncommon for different AI tools – such as reviewer matchers or novelty detectors – to produce divergent outputs for the same manuscript, particularly when small textual variations or formatting differences are introduced. Absent standardized benchmarks and validation protocols, reliance on these tools may yield divergent or even contradictory editorial outcomes. Editors bear the responsibility of critically evaluating the tools they deploy and resisting the temptation to treat algorithmic outputs as neutral or infallible.

Equally vital is the question of transparency. If editors are unable to interrogate the rationale behind an AI system’s recommendation, be it to reject a submission, route it for review, or select a reviewer, they risk delegating core scholarly judgments to mechanisms whose reasoning is opaque and potentially biased. Editorial oversight must therefore remain decisively human, even as it is augmented by technical assistance.

Ethical risks concern the societal consequences associated with the use of AI in scientific writing and publishing. A central ethical obligation for editors is the duty of informed consent. Authors and reviewers must be notified when AI systems are used to process their submissions or reviews, whether for screening, triage, or evaluation. Transparency allows authors to understand the basis on which their work is being assessed and to contest decisions that appear misaligned with the scientific content or intent of their manuscripts.

Another pressing ethical issue is data privacy. Manuscripts and reviewer reports may contain sensitive information, including unpublished data, confidential assessments, or proprietary methodologies. When this content is processed or stored by third-party AI vendors, especially those operating outside robust regulatory frameworks, questions arise about data security, intellectual property, and compliance with institutional and national data protection laws.

Authors also bear ethical responsibilities, particularly as generative AI tools become increasingly integrated into the research and writing process. Undisclosed reliance on AI to draft manuscripts or respond to reviewer feedback raises concerns about authorship integrity. Issues of originality, intellectual contribution, and accountability come to the fore when the boundaries between human and machine-generated content are not clearly marked. Authors must accept that transparency is not merely a formality but it is foundational to the ethics of scholarly authorship.

## Toward an ethical editorial framework for AI use

Both epistemic and ethical risks introduced by AI in scientific writing and publishing are addressed – explicitly or implicitly – within established professional standards and codes of conduct. The Committee on Publication Ethics (COPE), for instance, provides widely adopted guidelines on editorial transparency, authorship integrity, and ethical oversight, all of which are directly relevant to AI deployment [1, 2]. The International Committee of Medical Journal Editors (ICMJE) emphasizes accountability in editorial decisions, the disclosure of competing interests, and the integrity of peer review – standards that must now be reinterpreted in the context of algorithmic decision support [3, 4]. Publishers are also developing their own specific policies [5] while publishing alliances such as the STM Integrity Hub have begun developing collective responses to emerging threats, including the detection of manipulated images, paper mills, and inappropriate use of generative AI [6, 7].

These established frameworks provide a vital normative foundation for addressing the epistemic and ethical challenges introduced by AI. However, they remain largely high-level and are not yet tailored to the operational complexities of AI integration in scientific writing and publishing. Editorial teams require practical strategies to govern tool selection, ensure procedural fairness, and maintain scientific integrity within an increasingly algorithmically mediated landscape. To this end, we propose the following principles to support the responsible and accountable adoption of AI in scientific writing.

## A framework for editors: AI in editorial practice

- Human-in-the-loop design: AI systems should be designed to augment, not replace, human editorial judgment. Critical decisions – such as triage outcomes, reviewer selection, and final acceptance – must remain under the authority of human editors.
- Bias auditing and monitoring: Editorial teams should regularly assess AI tools for performance disparities across geography, discipline, institutional affiliation, and author demographics. Where disparities are identified, mitigation strategies should be implemented and documented.
- Tool provenance and validation: Only tools that have undergone rigorous validation via internal testing, peer-reviewed evaluation, or third-party audits should be deployed in editorial workflows. Selection should be based on demonstrable reliability, not convenience or cost.
- Transparency and disclosure: Authors, reviewers, and editorial board members should be notified when AI tools are used in manuscript processing, including triage, review facilitation, or content analysis. Disclosures should specify the nature and function of each tool used.
- Appeal and redress mechanisms: Journals should establish clear procedures for authors to contest decisions influenced by AI, particularly when those decisions appear inadequately justified. These mechanisms are vital for maintaining procedural fairness.
- Training and capacity building: Editors should be equipped with the skills needed to interpret AI outputs critically, assess their limitations, and make informed judgments. Ongoing training should be considered part of editorial professional development.

## A framework for authors: AI in scientific writing

- Disclosure and transparency: Authors should clearly disclose any use of AI tools in manuscript preparation, including writing, editing, literature search, or summarization. Journal guidance should be provided as to where disclosure should occur within the manuscript.
- Human accountability: Use of AI does not diminish the author’s responsibility. All submitted content – regardless of how it was generated – must be owned, verified, and ethically endorsed by the named human authors.
- Integrity in attribution: AI tools must not be credited as authors. While they may support aspects of manuscript development, they do not meet criteria for authorship and cannot assume legal or ethical responsibility for published work.
- Data security and confidentiality: Authors should avoid submitting sensitive, unpublished, or proprietary content to AI tools, particularly those operating on cloud-based or third-party platforms, unless institutional or publisher guidelines explicitly permit it.
- Bias awareness and epistemic vigilance: Authors must evaluate suggestions for literature, terminology, or conceptual framing to avoid reproducing epistemic biases or inaccuracies inherent in training data.
- Skill development and reflective practice: Engagement with AI tools should be informed and thoughtful. Authors, especially early-career researchers, should seek training and institutional support to use AI in ways that enhance, rather than undermine, scientific rigor and integrity.

Together, these principles articulate a shared responsibility for shaping the role of AI in scientific publishing. They are not intended to stifle innovation but to ensure that its adoption reinforces the foundational values that give scientific communication its trustworthiness and legitimacy.

### Note on tool selection

The AI tools referenced in this article are intended as illustrative examples and do not represent an exhaustive inventory of all technologies available at the time of writing. Selection was guided by the tools’ visibility within the scientific publishing community and accessibility to a broad range of users. Inclusion does not imply endorsement, nor does omission suggest lack of merit. None of the authors has any personal or financial connection with the companies or organizations that develop the AI-tools mentioned. As the landscape of AI in publishing is rapidly evolving, this list should be understood as a snapshot rather than a definitive catalogue.
